# Comment on “Prognostic value of middle paraesophageal lymph node metastasis in patients with pathologic N+ esophageal squamous cell carcinoma”

**DOI:** 10.1097/JS9.0000000000003261

**Published:** 2025-08-25

**Authors:** E. Dexiu

**Affiliations:** Department of Thoracic Surgery, Qinghai Provincial People’s Hospital, Xining, Qinghai, China

*Dear Editor,*

The recent study by Jiang *et al*^[^1^]^ provides valuable insight into the prognostic relevance of lymph node station 108 metastasis in patients with pathologic N+ esophageal squamous cell carcinoma (ESCC) undergoing upfront esophagectomy. The authors demonstrated that middle paraesophageal lymph node (station 108) involvement is significantly correlated with deeper tumor infiltration and represents an independent predictor for overall survival (OS), disease-free survival (DFS), locoregional recurrence, and distant relapse. This work highlights an often-underemphasized aspect of esophageal lymphatic dissemination.

The esophagus possesses a highly intricate lymphatic drainage system. While longitudinal submucosal lymphatics are well recognized for facilitating skip metastases, the role of intermuscular and paraesophageal drainage routes is less defined. Jiang *et al*’s observation that station 108 metastasis is strongly associated with advanced pT stage supports the anatomical basis that paraesophageal nodes reflect tumor penetration beyond the submucosa into deeper muscular layers. Currently, the 8th edition AJCC staging system considers only the number of metastatic lymph nodes, without regard to anatomical distribution^[^2^]^. However, emerging data, including this study and previous work by Tachimori and Miyata, indicate that metastatic location, particularly in paraesophageal and celiac zones, may hold independent prognostic significance^[^3,4^]^. We echo the authors’ call for greater attention to lymph node station-specific patterns in ESCC, which may eventually guide more tailored lymphadenectomy strategies.

While the findings are thought-provoking, several methodological limitations warrant discussion. First, the exclusion of patients who received neoadjuvant therapy, now widely regarded as standard of care for locally advanced ESCC, limits the generalizability of the findings. It remains unclear whether the prognostic value of station 108 metastasis persists after neoadjuvant chemoradiotherapy, especially given the potential for nodal downstaging and fibrosis that can obscure pathological assessment. Additionally, important pathological covariates such as lymphovascular or perineural invasion were not included in the multivariate model, which could confound the observed associations. An important analytical limitation is the treatment of station 108 metastasis purely as a binary variable, which may obscure potential dose–response relationships. Given that the number of retrieved and positive lymph nodes at station 108 was recorded, a more granular analysis—stratifying patients into 0, 1, and ≥2 positive nodes—could offer greater prognostic resolution. Re-running the Cox proportional hazards models using this stratification and testing for trend would clarify whether increasing nodal burden at station 108 confers progressively worse outcomes. Such an approach would align with TNM staging principles and enhance the clinical interpretability and applicability of the findings. Finally, the lack of imaging correlates and intraoperative visual references for station 108 hinders reproducibility across institutions and raises concerns about standardization in real-world practice.

Furthermore, with the increasing integration of artificial intelligence (AI) in surgical environments, future studies should explore how AI-driven tools—such as real-time decision support systems, predictive modeling, or intraoperative image-guided mapping—might facilitate precise nodal assessment, enhance intraoperative targeting, and support multidisciplinary coordination. The recently proposed TITAN guideline offers a standardized framework to ensure reproducibility and ethical integrity in AI-augmented surgical research and may serve as a foundation for future investigations seeking to embed AI within esophageal cancer workflows^[^5^]^.

In conclusion, this study delineates the prognostic significance of station 108 metastasis in ESCC. Incorporating this factor into surgical decision-making may enhance patient stratification, while further investigations are warranted to optimize lymph node assessment techniques and improve clinical outcomes.

## Data Availability

Not applicable.

## Research registration unique identifying number (UIN)

As a commentary, not applicable for research register.
